# Quantification of the Rupture Potential of Patient-Specific Intracranial Aneurysms under Contact Constraints

**DOI:** 10.3390/bioengineering8110149

**Published:** 2021-10-21

**Authors:** Manjurul Alam, Fernando Mut, Juan R. Cebral, Padmanabhan Seshaiyer

**Affiliations:** 1 Department of Bio-Engineering, George Mason University, Fairfax, VA 22030, USA; fmut@gmu.edu (F.M.); jcebral@gmu.edu (J.R.C.); 2 Department of Mathematical Sciences, George Mason University, Fairfax, VA 22030, USA; pseshaiy@gmu.edu

**Keywords:** intracranial aneurysms, hyper-elastic membrane, contact constraints, rupture potential, effective wall stress

## Abstract

Intracranial aneurysms (IAs) are localized enlargements of cerebral blood vessels that cause substantial rates of mortality and morbidity in humans. The rupture possibility of these aneurysms is a critical medical challenge for physicians during treatment planning. This treatment planning while assessing the rupture potential of aneurysms becomes more complicated when they are constrained by an adjacent structure such as optic nerve tissues or bones, which is not widely studied yet. In this work, we considered and studied a constitutive model to investigate the bio-mechanical response of image-based patient-specific IA data using cardiovascular structural mechanics equations. We performed biomechanical modeling and simulations of four different patient-specific aneurysms’ data (three middle cerebral arteries and one internal carotid artery) to assess the rupture potential of those aneurysms under a plane contact constraint. Our results suggest that aneurysms with plane contact constraints produce less or almost similar maximum wall effective stress compared to aneurysms with no contact constraints. In our research findings, we observed that a plane contact constraint on top of an internal carotid artery might work as a protective wall due to the 16.6% reduction in maximum wall effective stress than that for the case where there is no contact on top of the aneurysm.

## 1. Introduction

Intracranial aneurysms (IAs) are pathological enlargements of the cerebral arteries that have severe outcomes when they rupture [1,2,3,4,5]. Although most of these aneurysms do not rupture during a person’s lifetime, ruptured aneurysms result in subarachnoid hemorrhage, which causes significant morbidity and mortality rates (25–50% mortality rate and around 64% long-term disability rate) [6,7,8,9,10,11,12]. Moreover, endovascular and surgical aneurysm interventions can have more complications [13]. Due to this reason, physicians and clinicians must always answer the tough question of whether to intervene in the aneurysm or to conservatively follow the aneurysm. Furthermore, some clinical information (prior rupture, family history, hypertension, and smoking), the patient’s age, and anatomical information (location and size of the aneurysms) present useful evidence to predict the natural history and rupture potential of brain aneurysms [14]. Earlier, the size of an aneurysm was the major predictor to assess its rupture risk, but it was later found that smaller sized aneurysms accounted for a considerable number of ruptures [15]. Current clinical decision and aneurysm risk assessment would greatly benefit from suitable biomechanical modeling to comprehend the underlying mechanism of the initiation, progression, and rupture potential of aneurysms. It is usually accepted that the initiation and progression of aneurysms are induced by blood flow with a gradual degradation of the arterial wall [16,17]. However, the factors involved in the initiation, growth, enlargement, and rupture of intracranial aneurysms remain inadequately recognized. Cebral et al. reported that the rupture risk prediction of aneurysms depends on anatomical, clinical, geometrical, hemodynamic, and biomechanical information [16]. Furthermore, it is also important to connect wall biomechanical stress, strength, and failure analysis in aneurysms in conjunction with anatomical, clinical, and flow characteristics. Moreover, the rupture potential in cases when the aneurysms are symptomatic (constrained by contact constraints such as nerve tissues) might be different than when the aneurysms are asymptomatic (no contact constraints) [17,18,19]. Therefore, appropriate constitutive interaction modeling could help better understand the effect of contact constraining at the fundus of the aneurysm dome.

In this study, our main goal is to evaluate the variety of effective wall stress and of unruptured intracranial aneurysms to assess the rupture potential. To accomplish our purpose, we consider the clinical data of four patient-specific unruptured intracranial aneurysms (three middle cerebral arteries (MCAs) and one internal carotid artery (ICA)). We study these data with biomechanical models using appropriate constitutive equations and knowledge from cardiovascular solid mechanics.

## 2. Materials and Methods

### 2.1. Patient Clinical Information

The clinical summary of the data that were employed is presented in Table 1, including anatomical information of the patients such as age, gender, family history of aneurysms, and lifestyle information including cigarette smoking, hypertension, and diabetes status of the patients [20]. CT scan was used to evaluate the number of aneurysms, subarachnoid hemorrhage (SAH) status, and to locate the aneurysms. The patients who were presently being treated with hypertension and the patients who were previously diagnosed but not being treated were counted as hypertensive patients. The patients who presently smoked or smoked within the last five years were counted as smokers, and smoking was registered as packs of cigarettes per week.

The mechanical properties used in the simulation are presented in tabular form in Table 2 for different aneurysm cases. A uniaxial loading system compatible with an Olympus FV1000 MPE multiphoton microscope was employed on these different aneurysm samples to test them for different mechanical parameters. To test the samples, they were first gripped by a mechanical clamp and subjected to uniaxial tension at a speed of 20 μm/s along the circumference of the arteries. Using a linear actuator (ANT-25 LA) and a 2.27-kg load cell (MDB-5, Transducer), displacement and force were recorded, respectively. After plotting the displacement–force curve for five cycles with a preconditioning load of 0.3 N, the Cauchy stress was calculated as a function of strain [20].

### 2.2. Governing Model Equations

In this work, structural modeling of patient-specific intracranial aneurysms was performed with the help of an uncoupled Mooney–Rivlin model and with its material parameters. This model is an uncoupled deviatoric hyper-elastic model with volumetric behavior and a phenomenological type. The Mooney–Rivlin model describes the material behavior as a function of different strain invariants with parameters (two, three, five, or nine) depending on the stress–strain curve of the material [21,22,23,24,25].

The stress state for a given strain is calculated as the derivative of the strain energy density. The strain energy density function can be written as follows:

(1) $$W=W\left({\bar{I}}_{1},{\bar{I}}_{2},J\right)=W\left({\bar{I}}_{1},{\bar{I}}_{2}\right)+U\left(J\right)$$

Second, Piola–Kirchhoff stress is given as follows:

(2) $$\bar{S}=\frac{\partial \bar{W}\left({\bar{I}}_{1},{\bar{I}}_{2}\right)}{\partial E}$$

The strain energy function for the Mooney–Rivlin model can be written as follows:

(3) $$W=C_{1}\left({\bar{I}}_{1}-3\right)+C_{2}\left({\bar{I}}_{2}-3\right)+\frac{1}{2}K{\left(lnJ\right)}^{2}$$

where $C_{1}$ and $C_{2}$ are the Mooney–Rivlin material coefficients; ${\bar{I}}_{1}$ and ${\bar{I}}_{2}$ are the first and second invariants of the deviatoric right Cauchy–Green deformation tensor, respectively; K is the bulk modulus-like penalty parameter; and J is the determinant of the deformation gradient tensor. The Mooney–Rivlin model reduces to an uncoupled neo-Hookean model when the coefficient $C_{2}=0$. This material model interpolates displacements as linear field variables and the volume ratio and pressure as piecewise constants for each element, consisting of a three-field element formulation [25].

The principal first and second invariants ${\bar{I}}_{1}$ and ${\bar{I}}_{2}$ can be written as follows:

(4) $${\bar{I}}_{1}=\lambda_{1}^{2}+\lambda_{2}^{2}+\lambda_{3}^{2},\;{\bar{I}}_{2}=\lambda_{1}^{2}\lambda_{2}^{2}+\lambda_{2}^{2}\lambda_{3}^{2}+\lambda_{3}^{2}\lambda_{1}^{2}$$

where $\lambda_{i}$ is assumed as constant for each deformed configuration.

Considering an isotropic, incompressible hyper-elastic membrane, non-zero Cauchy stress can be written as follows [26]:

(5) $$\begin{array}{c} t_{11}=-p+2W_{1}\lambda_{1}^{2}-2W_{2}\lambda_{1}^{-2}\\ t_{22}=-p+2W_{1}\lambda_{2}^{2}-2W_{2}\lambda_{2}^{-2}\\ t_{33}=-p+2W_{1}\lambda_{3}^{2}-2W_{2}\lambda_{3}^{-2} \end{array}$$

where *p* is the Lagrange multiplier, $W_{1}=\frac{\partial W}{\partial {\bar{I}}_{1}}$ and $W_{2}=\frac{\partial W}{\partial {\bar{I}}_{2}}$ are the response functions, and $t_{ii}$ represents the Cauchy stress.

Then, the effective stress tensor can be written in terms of Cauchy stress tensor as follows [27]:

(6) $$\bar{t}=M:t$$

where M is the overall damage effect tensor, which is a symmetric fourth-rank tensor. The effective or von Mises stress is a stress that takes both normal and shear stresses into account while measuring the local maximum stress.

A plane contact constraint (nerve contact) was introduced on the aneurysm fundus, which was considered to be uniform, rigid, and stationary. The plane contact constraint (nerve contact) used in the simulation was assumed as an elastic material with an elastic modulus of 40.96 ± 2.59 MPa and a Poisson ratio of 0.37 ± 0.02 [28,29,30]. The average density of nerve tissue was measured from 1.020 to 1.035 g/cm^3^ [31]. Tied facet-to-facet contact was utilized for interaction between the aneurysm fundus wall and planar nerve, where the planar nerve is considered a master surface and the aneurysm fundus wall as a slave surface.

To satisfy multipoint constraint, we can write the following:

(7) $$A_{1}u_{i}^{X}+A_{2}u_{j}^{Y}+A_{N}u_{k}^{Z}=0$$

### 2.3. Computational Simulation

**Figure 1:**
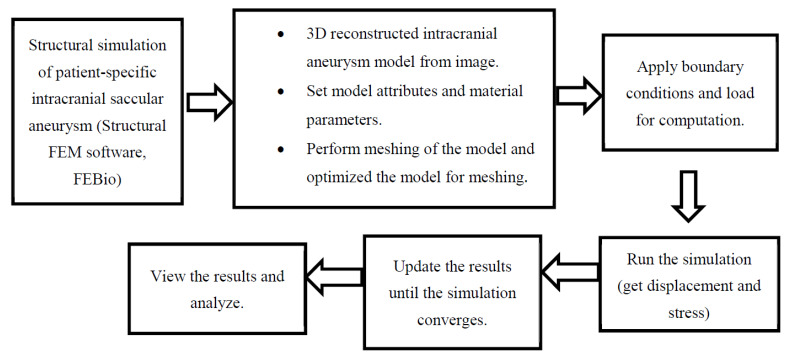
Steps of finite element simulation of unruptured intracranial aneurysms in FEBio.

To perform structural simulation of the patient-specific intracranial saccular aneurysms, we first reconstructed a 3D intracranial aneurysm model from 3D angiography images. Setting model attributes and material parameters to the computational geometry, we performed meshing for the model. In the simulation, meshing was performed by using a tetrahedral mesh. The mesh edge function was used to handle the mesh density of the geometry. A mesh convergence study was performed to find the optimum number of nodes and elements needed for the simulation. A steady-state finite element simulation approach was utilized in this work to simulate the patient-specific unruptured intracranial aneurysms using the open-source structural finite element software package FEBio [21]. The extracted 3D geometric reconstruction was imported into the finite element software for the required meshing. Then, after applying load and appropriate boundary conditions, the model was run for the simulation. The aneurysm wall thickness and material properties of the wall were considered uniform throughout the simulation process. The force applied to the aneurysm wall was assumed to be a uniform systolic pressure force of 120 mmHg, working from inwards to the outward direction of the aneurysm. The process of modeling and simulation of unruptured intracranial aneurysms using open-source finite element software FEBio is illustrated in Figure 1 in a flowchart.

### 2.4. Boundary Conditions and Loads

Two boundary conditions (displacement and traction) were applied in the geometry to perform the simulation. Considering the computational domain in the current configuration $\beta_{t}$ as Ω and the surface of Ω as ∂Ω, we can write the following.

Displacement boundary condition:

(8) $$u=\bar{u}\;\mathrm{on}\;\partial \Omega_{u}$$

where u is defined as u=x−X.

Traction boundary condition:

(9) $$T^{\left(n\right)}={\bar{T}}^{\left(n\right)}\;\mathrm{on}\;\partial \Omega_{T}$$

where $T^{\left(n\right)}=n.t$ and $\partial \Omega =\partial \Omega_{u}\cup$ $\partial \Omega_{T}$.

A uniform systolic pressure (P=120 mmHg) force was assumed and applied inside the aneurysm from inward to the outward direction throughout the simulation.

## 3. Results

**Figure 2:**
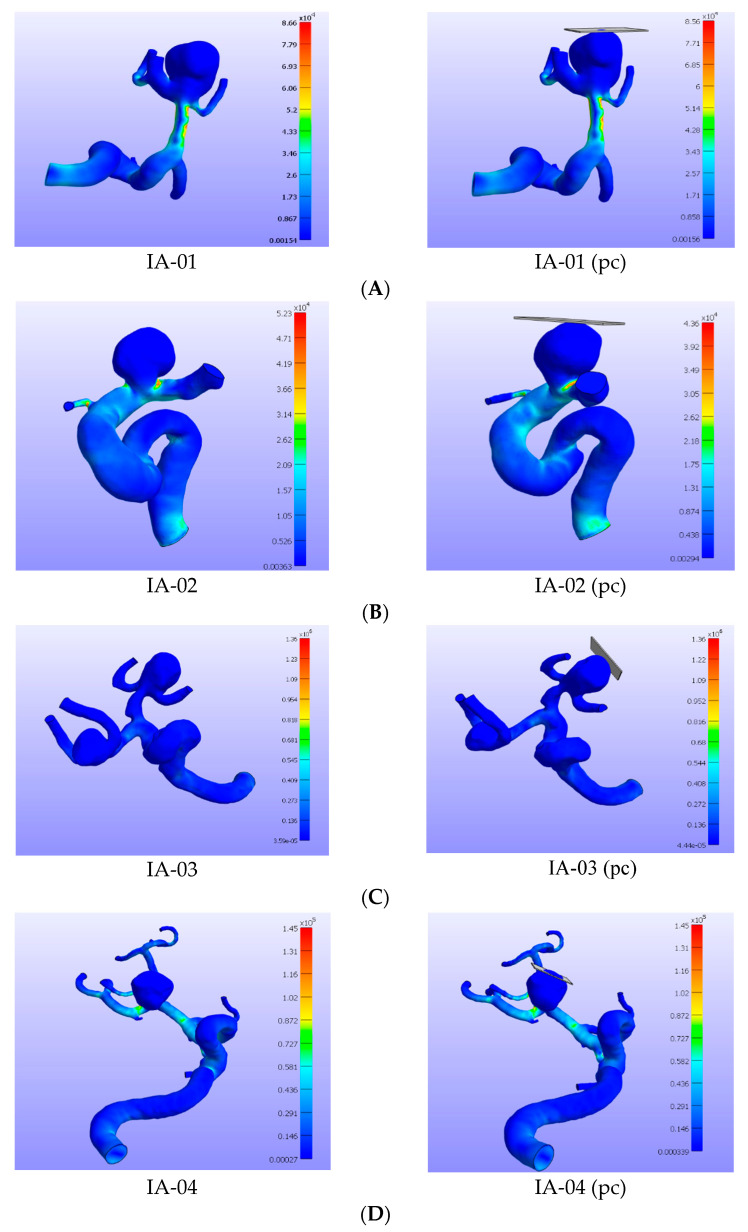
Finite element simulations of effective stress (Pa) on patient-specific unruptured intracranial aneurysms, where the panels on the left are aneurysms without constraint and on the right-side are the respective aneurysms with plane contact (pc) constraint for each patient. The simulations were performed under a uniform internal pressure of 120 mmHg. The cases IA-01 (**A**), IA-03 (**C**), and IA-04 (**D**) are middle cerebral artery aneurysms, and IA-02 (**B**) is an internal carotid artery aneurysm. The panels for (**A**) and (**B**) illustrate that maximum effective stress was decreased more when the aneurysm was constrained by a plane contact than that in the cases of an aneurysm with no contact. No noticeable change in maximum effective wall stress for the third (**C**) and fourth (**D**) panels were observed between the aneurysm without contact and the aneurysm with plane contact.

The rupture potential of an aneurysm can be estimated when the maximum wall effective stress exceeds the maximum wall strength at any spatial point of the aneurysm. The effective wall stress for four different intracranial aneurysms is shown in Figure 2 for the cases where there was no contact constraint and where there was a plane contact constraint. From the first two simulation results (IA-01 and IA-02), it was seen that aneurysms with plane contact constraint produced less maximum effective wall stress (0.0866–0.0856 MPa (1.15% reduction for IA-01) and 0.0523–0.0436 MPa (16.6% reduction for IA-02)) compared to when there was no constraint. The maximum effective wall stress was also found at or near bifurcation areas of the aneurysms for all the aneurysm cases. On the other hand, the simulation results from the other two aneurysms (IA-03 and IA-04) suggested that there was almost no change in effective wall stress (1.36 MPa for IA-03 and 0.145 MPa for IA-04) when plane contact constraint was present on top of the aneurysm compared to when there was no contact constraint.

**Figure 3:**
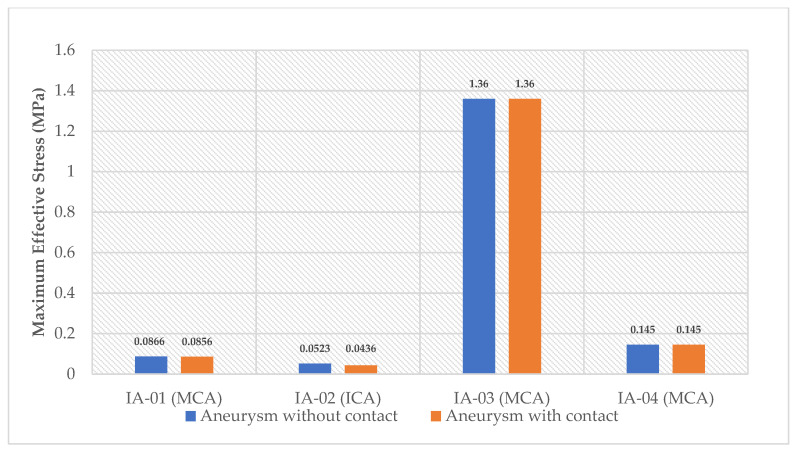
Comparison of maximum effective stress (MPa) of patient-specific intracranial aneurysms between cases without contact constraint and cases with plane contact constraint.

The comparison of the maximum effective stress of unruptured intracranial aneurysms between aneurysms with plane contact constraint and aneurysms without contact constraint is shown in Figure 3. From the chart, we can see that the maximum effective stress for the first two cases was decreased more when the aneurysm was constrained by a plane contact than that in the cases of an aneurysm with no contact. A decrease of 1.15% occurred for the case IA-01 (MCA), whereas a decrease of 16.6% happened for the case IA-02 (ICA). Conversely, there was no noticeable change in maximum effective wall stress for the other two aneurysm cases (IA-03 and IA-04) between the aneurysm without contact and the aneurysm with plane contact. By observing the effective wall stress output, it can be predicted that the plane contact constraint on top of the IA-02 aneurysm (ICA) might provide some protection for the aneurysm, whereas the rest of the aneurysms might not show any protective effect.

As IA-02 (ICA) showed a higher reduction in maximum effective wall stress than that of any other aneurysms, it can be assumed that IA-02 (ICA) might provide some protection due to the presence of plane contact constraint, showing lower risk of rupture.

## 4. Discussion

If an intracranial aneurysm is balanced by an adjacent structure (such as optic nerve tissue), it might be worth not performing surgery immediately; on the other hand, if it is not balanced by an adjacent structure, it might be necessary to plan for an immediate step. In this work, the biomechanical Mooney–Rivlin constitutive model was used to simulate, quantify, and predict the rupture potential of unruptured intracranial aneurysms under plane contact constraints. Based on the two simulation parameters (maximum effective wall stress and maximum total displacement), we analyzed and predicted the effect of plane contact constraint on the four patient-specific unruptured intracranial aneurysms. From the simulation and analysis, we observed that the second aneurysm case IA-02 (internal carotid artery) with plane contact constraint showed a 16.6% reduction in maximum effective wall stress compared to the case where IA-02 had no contact constraint. On the other hand, the IA-01, IA-03, and IA-04 middle cerebral aneurysms did not show any reduction in effective maximum wall stress. This might be due to the fact that the geometrical structure of the aneurysm IA-02 (ICA) might provide the plane contact constraint a better chance to absorb some load from the aneurysm at the top and gradually down to the bottom where maximum effective wall stress occurred at the neck. This result showed a slight indication to prove an earlier hypothesis that a plane contact constraint on top of a simple, axisymmetric aneurysm reduced Cauchy stresses of the aneurysm and provided slight protection or was at least not harmful due to the balanced constraint [17,18,19]. However, for the cases IA-01, IA-03, and IA-04, the plane contact constraint did not provide enough support to take some load from the aneurysm fundus to the neck because of the shape, orientation, and geometrical complexity.

## 5. Conclusions

The results obtained from the study need to be validated with larger samples of patient-specific data to obtain better insights into the hypothesis. Moreover, the simulation performed in this work is based upon a structural hyper-elastic wall modeling of aneurysms using cardiovascular structural equations. In this work, the simulations were performed by assuming the plane contact constraint to be rigid and stationary and in contact with the aneurysms. This is a specific case where aneurysms were in contact with the surrounding tissues or structures. If the surrounding tissues are not fixed or stationary, then time-dependent contact constraint cases are necessary. For our simulation, stationary contact constraint alone was enough to model the contact between aneurysms and adjacent structures. Although the results from this simulation provide a good indication or insight for aneurysms constrained by contact for specific patient cases, it is important to work further in this area combining fluid–structure contact modeling by considering more parametric analyses such as the size and shape of the contact, the orientation of the contact, changes in the size and shape of the aneurysm due to growth and remodeling, etc.

## Figures and Tables

**Table 1:** Clinical data summary for four unruptured intracranial aneurysm cases (MCA = middle cerebral artery, ICA= internal cerebral artery) [20].

Aneurysm	Location of IA	Gender	Age	SAH	Family History of IA	Number of IA	Cigarette Smoker (Packs/Week)	Hypertension	Diabetic
IA-01	MCA	F	61	No	Yes	1	Yes (10)	Yes	No
IA-02	ICA	M	63	No	No	2	No	Yes	Yes
IA-03	MCA	F	69	No	No	2	Yes (1)	No	No
IA-04	MCA	F	66	No	No	1	Yes (7)	Yes	No

**Table 2:** Mechanical parameters of the unruptured intracranial aneurysms [20].

Aneurysm Domes	Ultimate Stress (MPa)	Ultimate Stretch	Young’s Modulus, E (MPa)	$\mathrm{Material}\;\mathrm{Parameter},\;c_{1}\;(\mathrm{MPa})$	$\mathrm{Material}\;\mathrm{Parameter},\;c_{2}$	Bulk Modulus, K (MPa)	Poisson’s Ratio (ϑ)
IA-01	1.50	1.27	1.18	5.47	1.10	1.967	0.3
IA-02	0.625	1.24	0.50	3.76	0.54	0.834	0.3
IA-03	0.73	1.05	0.70	338.13	0.54	1.166	0.3
IA-04	1.34	1.33	1.008	1.45	8.38	1.679	0.3

## Data Availability

No new data were created or analyzed in this study. Data sharing is not applicable to this article.
